# Impact of Sound and Music on Cancer Cell Viability—Current Evidence and Mechanistic Insights

**DOI:** 10.3390/cimb48101088

**Published:** 2026-10-21

**Authors:** Wolfgang Jungraithmayr

**Affiliations:** Division of Thoracic Surgery, Rostock University Medical Center, Schillingallee 35, 18057 Rostock, Germany; wolfgang.jungraithmayr@med.uni-rostock.de

**Keywords:** sound, music, cancer cells, viability

## Abstract

Music therapy is established in oncology as a supportive intervention to reduce anxiety, pain, distress and treatment-related symptom burden. Beyond these clinical effects, emerging experimental evidence suggests that audible sound and music may also exert direct biological effects on non-auditory cells, including cancer cells. This review summarizes current evidence on the effects of audible sound, music and acoustic stimulation on cancer-cell viability and related cellular processes. Sound represents a mechanical pressure wave that may interact with cells through mechanotransductive processes involving the plasma membrane, cytoskeleton, integrins, focal adhesions and mechanosensitive ion channels. Since cancer cells frequently display altered stiffness, cytoskeletal organization, membrane dynamics and mechanosensitivity, they may respond differently to acoustic or vibrational stimulation than non-malignant cells. Available in vitro studies report associations among audible sound, music, acoustic stimulation and cancer-cell viability, proliferation, migration, apoptosis-related signalling, gene expression and extracellular vesicle secretion. These effects have been demonstrated in breast, gastric, prostate, glioblastoma, colon, ovarian and neuroblastoma cell models, but findings appear highly cell-type-specific and dependent on exposure parameters. Proposed mechanisms include intracellular calcium signalling, cytoskeletal remodelling, mitochondrial dysfunction, reactive oxygen species generation and pathways involving PIEZO1, integrins, focal adhesion kinase and YAP/TAZ; however, causal mechanisms are not shown. Much of the mechanistic framework derives from ultrasound-based research and cannot be extrapolated to audible sound or music. Overall, the reported findings of in vitro experiments on the effect of sound on cancer cells remain preliminary, methodologically heterogeneous and do not establish reproducible, causal or cancer-selective effects. Standardized studies are required before these observations can be translated into mechanistically defined or therapeutically relevant concepts in oncology.

## 1. Introduction

Cancer remains one of the leading causes of mortality worldwide despite major advances in diagnostics and therapies. Complementary therapeutic approaches that have the potential to improve cancer treatment outcome have gained increased attention in recent years. Among these emerging areas, the biological effects of music and acoustic stimulation on early developmental stages of cancer have attracted growing scientific interest. While music therapy has long been employed in oncology to reduce anxiety, pain, and psychological distress in patients [1], emerging research suggests that music and acoustic stimuli may also exert direct effects on cellular physiology, including cancer-cell viability and proliferation [2,3].

Traditionally, the effects of music on humans have been understood primarily through neuropsychological and emotional pathways mediated by the auditory system. Early observations from the biologist Helene Grimal and Fabien Maman from the 1980s, however, studied the relationship of sound waves to living cells. With a camera mounted on a microscope, they observed that uterine cancer cells exposed to different acoustic instruments such as guitar, gong, xylophone as well as the human voice and found that cancer cells lost structural integrity [4]. Despite remarkable findings, this experimental study was an unverified anecdotal description without documented controls or acoustic measurements and no quantitative outcome. The first experimental, peer-reviewed studies published stem from Lestard and colleagues, who reported cellular changes after music or sound exposure in vitro [2,3]: in their studies, exposure to different musical compositions altered the viability, migration, and metabolic activity of cultured human cancer-cell lines. However, these observations did not identify which acoustic variables are responsible or demonstrate a direct causal biomechanical mechanism.

From a biophysical perspective, music corresponds to vibrations transmitted in the form of pressure waves. Cells are sensitive to mechanical stimulation and continuously interact with their physical microenvironment through mechanotransduction [5,6]. Vibrations can influence cytoskeletal organization, membrane permeability, ion channel activation, intracellular signalling pathways, and gene expression [5]. Importantly, cancer cells often exhibit altered mechanical properties compared with healthy cells, such as structural changes, cytoskeletal disorganization, and abnormal membrane dynamics [6,7]. These differences raise the hypothesis that malignant cells may respond differently to acoustic or vibrational stimulation than non-malignant cells.

Beyond music exposure itself, related research fields have explored the effects of mechanic pressure waves on tumour cells, for example ultrasound and resonance-based therapies [8,9]. Experimental approaches such as focused ultrasound [9] and the theoretical concept of “oncotripsy” suggest that cancer cells may possess unique mechanical resonance frequencies that could potentially be exploited for selective therapeutic targeting.

Despite the heterogeneity of parameters from existing studies, such as experimental conditions, cancer models, and outcome measurements, the observed interactions between sound-induced mechanical changes and tumour-cell biology represent a scientifically relevant area worthwhile of systematic examination.

The aim of this review is therefore to summarize and critically evaluate the current experimental evidence regarding the effects of music, sound frequencies, and acoustic stimulation on cancer-cell viability. In addition, we discuss potential mechanobiological mechanisms underlying these effects, highlight major methodological limitations within the field, and outline future directions for research at the intersection of acoustics, mechanotransduction, and oncology.

## 2. Review Type and Search Strategy

This is a narrative review. Relevant publications were selected to discuss experimental effects of audible sound or music on cancer cells and to place proposed mechanisms in the context of related acoustic and mechanobiological studies. The sources searched were Embase, Pubmed, Web of Science, and Scopus, using the search terms sound, music, cancer, viability, and mechanotransduction, with the last search on 26 August 2026. No formal risk-of-bias or study-quality assessment was performed; the findings were therefore described as limited and methodologically heterogeneous rather than assigned a formal evidence grade.

## 3. Biological Mechanisms of Sound–Cell Interactions

Sound is a mechanical form of energy that propagates through a medium as oscillating pressure waves. Depending on their frequency, amplitude, and duration, sound waves can induce physical forces capable of interacting with biological tissues and individual cells.

Sound is not only subject to auditory perception but may directly influence cellular behaviour through biomechanical and biochemical mechanisms independent of the nervous system [6,7]. At a cellular level, mechanical stimuli are converted into biochemical signals through a process known as mechanotransduction. Cells continuously sense and respond to the physical properties of their microenvironment, including pressure, stiffness, tension, and vibration [6]. This process is mediated by various cellular and membrane structures such as integrins, adhesion complexes, stretch-activated ion channels, the extracellular matrix, and the cytoskeleton [6,7,10]. Mechanical forces transmitted through these structures can therefore induce cell membrane deformation, alter cytoskeletal organization, intracellular signalling pathways, gene expression, membrane permeability, and remodels extracellular matrix (ECM). Particularly the cytoskeleton plays a central role in cellular mechanosensing. The cytoskeleton consists primarily of actin filaments, microtubules, and intermediate filaments that maintain structural integrity and facilitate intracellular force transmission. Acoustic vibrations may generate oscillatory mechanical stress within these structures, potentially influencing cell morphology, motility, and survival. Baumgartner and colleagues recently showed in an in vitro wound healing model that mechanic vibration alters the actin cytoskeleton, leading to a more directed and accelerated cell migration with a higher filament alignment along the wave’s propagation axis in surface acoustic wave wound healing [11].

Mechanical deformation of the plasma membrane can additionally activate mechanosensitive ion channels by the application of force to integrins, resulting in calcium influx into the cytoplasm which may modulate contractility through calmodulin–caldesmon interactions and thereby actively influence cytoskeletal organization [12]. An important mechanosensitive ion channel is Piezo1. Activation of Piezo1 results in the influx of ions such as Ca^2+^, thus triggering multiple signalling pathways such as PI3K/Akt and membrane type 1-matrix metalloproteinase (MT1-MMP) [13,14]. This is implicated in processes like vascular remodelling and angiogenesis and downstream signalling cascades associated with proliferation, stress responses, and apoptosis. Interestingly, there is an interaction between Piezo1 and the cytoskeleton: the loss of the actin cytoskeleton makes Piezo1 more sensitive and easier to activate. Thus, the cytoskeleton acts as a membrane tension regulator, playing a mechanoprotective role and at the same time affecting Piezo1 activity [15].

## 4. General Mechanisms of Sound–Cancer-Cell Interactions

Structural changes in the cytoskeletalon are highly relevant in cancer because cytoskeletal plasticity controls migration, invasion, epithelial–mesenchymal transition and resistance to mechanical stress. Cancer cells are known to possess altered biomechanical properties and mechanosensitivity compared with healthy cells. Malignant cells often exhibit reduced stiffness, cytoskeletal disorganization, altered adhesion dynamics, and increased deformability [7,16,17]. These characteristics contribute to tumour progression and metastatic potential but may also increase susceptibility to external mechanical stimuli. Several studies have therefore proposed that cancer cells may respond differently to acoustic or vibrational energy than non-malignant cells [18,19,20,21,22]. Mechanosensitive signalling pathways involving FAK, YAP/TAZ, integrins, and PIEZO channels play central roles in cancer-cell adaptation to mechanical stress. Mechanical and ultrasound studies suggest that oscillatory forces may engage these pathways, but equivalent causal activation by audible music has not been established. One proposed mechanism involves resonance. Resonance occurs when an external vibration matches the natural oscillatory frequency of a structure, thereby amplifying mechanical motion. The theoretical concept of “oncotripsy,” introduced by Heyden and Ortiz, suggests that cancer cells may possess unique resonance frequencies due to differences in cellular geometry, elasticity, and cytoskeletal architecture [23]. Dynamical models demonstrated that harmonic excitation at specific ultrasonic frequencies could selectively disrupt malignant cells while sparing healthy tissue [18].

The strongest evidence exists for ultrasound-based interventions. Here, intracellular calcium oscillations are among the reported early responses. Ultrasound-generated mechanical forces can deform the plasma membrane and activate mechanosensitive channels such as PIEZO1, thereby promoting calcium influx. In tumour cells, this calcium entry has been linked to downstream activation of calpain, mitochondrial dysfunction and apoptosis. Tijore et al. showed that ultrasound-mediated mechanical forces selectively induced apoptosis in tumour cells through a calcium-dependent, PIEZO1-associated and calpain-mediated mitochondrial pathway, while normal cells were less susceptible [24]. Calcium oscillations also serve as signalling hubs that connect acoustic stimulation with cytoskeletal dynamics, mitochondrial activity and transcriptional regulation, making Ca^2+^ signalling one of the most important interfaces between acoustic physics and cancer-cell behaviour [25].

Another major response to acoustic stimuli involves mitochondrial signalling. Calcium influx induced by acoustic stimulation can be taken up by mitochondria, where it alters mitochondrial membrane potential, oxidative phosphorylation and apoptotic sensitivity. In sonodynamic therapy, mitochondria are frequently described as primary subcellular targets because many sonosensitizers accumulate in or near mitochondria. Upon ultrasound exposure, activated sonosensitizers generate reactive intermediates that damage mitochondrial membranes, reduce mitochondrial membrane potential, promote mitochondrial swelling and activate caspase-dependent apoptosis. A recent comprehensive review on nanosensitizer-assisted sonodynamic therapy in breast cancer summarizes that ultrasound-induced ROS can damage the mitochondrial membrane, decrease mitochondrial membrane potential, increase caspase-3 and caspase-9 activation, and induce apoptosis in breast cancer cells [26].

The generation of reactive oxygen species (ROS) represents a key biochemical bridge between acoustic energy and cancer-cell death. ROS may arise from cavitation, sonoluminescence, sonosensitizer activation, mitochondrial electron transport disruption and inflammatory cell responses. At moderate levels, ROS can act as secondary messengers; at excessive levels, they contribute to mitochondrial outer membrane permeabilization (MOMP), a critical event in the intrinsic apoptotic pathway, leading to activation of BAX/BAK proteins, cytochrome c release, and caspase-mediated cell death. This is particularly important in cancer cells, which often operate near a redox threshold because of oncogene-driven metabolism and chronic oxidative stress. Sonodynamic therapy deliberately exploits this vulnerability: low-intensity ultrasound activates sonosensitizers to produce ROS, which then induce mitochondrial dysfunction, apoptosis, ferroptosis, autophagy modulation and immunogenic tumour-cell death [27].

**Figure 1:**
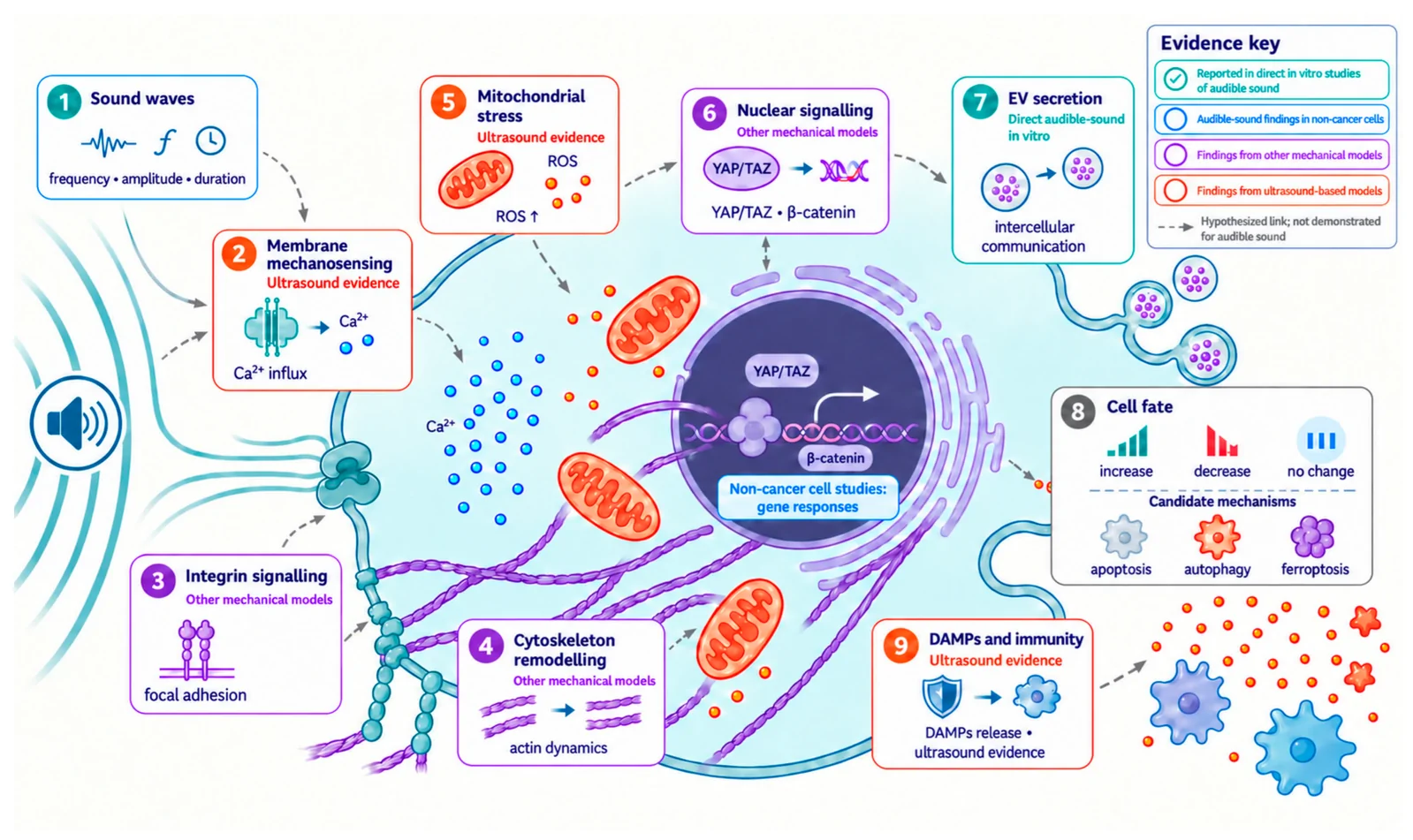
Evidence and proposed mechanisms concerning the effects of music and audible sound on cells. Direct in vitro studies of audible sound or music have reported changes in cancer-cell viability, migration, apoptosis-related markers and extracellular vesicle (EV) secretion. Changes in viability may be positive, negative or absent, depending on the cell model and exposure conditions. Blue identifies gene responses reported after audible-sound exposure in non-cancer cells; purple identifies findings from other mechanical models. Orange identifies mechanisms investigated mainly in ultrasound-based models, including PIEZO1-associated Ca^2+^ influx, mitochondrial stress, reactive oxygen species (ROS) production, damage-associated molecular pattern (DAMP) release and immune activation. These findings must not be assumed to occur under audible-sound exposure. Grey dashed arrows indicate hypothesized links, not demonstrated causal steps. In particular, a continuous sequence from audible sound to Ca^2+^ influx, mitochondrial stress, ROS production, cell death and immune activation has not been established. Ca^2+^, calcium ion; TAZ, transcriptional coactivator with PDZ-binding motif; YAP, yes-associated protein.

These calcium-, cytoskeleton-, mitochondrial- and ROS-dependent events converge on apoptotic pathways. Focused ultrasound can influence apoptotic signalling through membrane permeabilization, calcium overload, mitochondrial outer membrane permeabilization, caspase activation and DNA damage [28]. The work by Tijore and colleagues particularly emphasizes that ultrasound can trigger selective cancer-cell apoptosis through PIEZO1-dependent calcium influx and calpain-mediated mitochondrial signalling [24] or targeting cancer-associated fibroblasts [29]. Ultrasound, histotripsy and sonodynamic therapy can promote membrane disruption, antigen release, DAMP exposure, cytokine signalling and recruitment or activation of dendritic cells, macrophages, and T cells [30]. Recent work on acoustic immune reprogramming describes ultrasound-responsive platforms as tools for spatially controlled modulation of the immune microenvironment, including induction of immunogenic cell death and remodelling of macrophage, dendritic-cell and T-cell functions [31]. Sonodynamic therapy can also induce immunogenic cell death through ROS generation and DAMP release, thereby linking direct tumour-cell killing with systemic anti-tumour immune activation [32]. Histotripsy provides an additional non-thermal acoustic strategy: by mechanically fractionating tumour tissue, it can release tumour antigens and DAMPs while preserving immunogenic material, thereby supporting local and abscopal immune responses. These mechanistic changes, particularly PIEZO1 activation, mitochondrial damage, ROS production and immune activation, are candidate mechanisms for audible-sound exposure, inferred largely from ultrasound studies; however, none is established as an effect of audible sound in cancer cells. Figure 1 summarizes the proposed underlying mechanisms.

## 5. Mechanistic Evidence for Audible Sound- and Music-Induced Effects on Cancer Cells

Compared with ultrasound-based approaches, the molecular mechanisms underlying the effects of audible sound and music on cancer cells are rather poorly defined. This distinction is important because many mechanisms commonly discussed in acoustic oncology—including PIEZO1-dependent Ca^2+^ influx, mitochondrial dysfunction, reactive oxygen species (ROS) generation, and immunogenic cell death—have been demonstrated predominantly under ultrasound, focused ultrasound, sonodynamic therapy, or histotripsy conditions and cannot currently be assumed to operate equivalently within the audible frequency range.

Nevertheless, experimental studies using audible acoustic stimulation provide evidence that mammalian cells can directly respond to sound in the absence of an auditory system. Controlled audible-sound exposure has been shown to alter mechanosensitive gene expression, with changes in genes such as PTGS2, CTGF, and TNC, indicating that audible acoustic energy can be translated into cellular biochemical responses [33,34]. These responses are dependent on acoustic parameters and cell type, supporting the concept that audible sound represents a weak but biologically detectable mechanical stimulus rather than a uniform cellular signal. Systematic evaluation of speaker-based cell-culture studies likewise indicates that cellular responses to audible sound are heterogeneous and highly dependent on experimental conditions [10].

For cancer cells specifically, the experimentally supported mechanisms are currently more restricted. Available studies report associations with apoptosis-related markers, cancer-related gene expression, cell migration and extracellular vesicle (EV) biogenesis or secretion. In particular, apoptosis-associated marker changes have been reported following music exposure, although the p53/caspase findings do not establish causality and thus require validation, while a controlled audible-wave study reported altered EV output alongside changes in ESCRT-, RAB-, SNARE- and RHO-associated trafficking markers [3,35]. These findings require confirmation and do not establish reproducible causal pathway activation or selectivity for cancer cells. Importantly, however, such responses are not uniformly against cancer; depending on the acoustic stimulus and cellular context, audible sound has also been associated with proliferative or pro-migratory responses.

Direct activation of PIEZO1-dependent Ca^2+^ influx, mitochondrial membrane dysfunction, ROS-mediated cytotoxicity, and immune-related signalling has not yet been convincingly demonstrated as a mechanistic sequence in cancer cells exposed specifically to audible sound or music. These pathways should therefore be regarded as candidate mechanisms derived largely from ultrasound and general mechanobiology rather than as validated mechanisms of audible-sound-induced anticancer activity. Similarly, vibration-induced modulation of YAP/β-catenin signalling provides supportive evidence for the mechanosensitivity of cancer cells but should be distinguished from direct music or audible-sound exposure. The experimental evidence underlying these aspects—including cancer-cell models investigated, musical or acoustic stimuli applied, exposure conditions, effects on cell viability and the induction of cancer-cell death—is discussed in detail in the following section.

## 6. Experimental Studies About the Effect of Music and Audible Sound on Cancer-Cell Viability and Cancer-Cell Death

Compared to ultrasound application, studies relating music or audible-sound exposure to cancer-cell viability remain limited and heterogeneous. The available evidence primarily relies on in vitro studies using loudspeaker-based exposure systems, different musical compositions, religious recitation, or audible vibration paradigms. These studies report changes in viability, proliferation, motility, gene expression and apoptosis-related markers after exposure, without establishing the acoustic cause or a shared mechanistic pathway. The evidence remains preliminary, and the field is limited by variability in sound intensity, frequency spectrum, exposure duration, distance from the sound source, culture conditions, and endpoint assays. To address this heterogeneity, the available studies should be interpreted according to recurring quality domain acoustic characterization of the stimulus, including sound-pressure level, frequency spectrum and waveform; (ii) reproducibility of exposure geometry and duration; (iii) use of sham-exposed and, where appropriate, non-malignant control cells; (iv) biological depth of the endpoints, distinguishing short-term metabolic viability assays from direct measures of apoptosis or long-term survival; and (v) independent replication. Across the literature, no study fulfils all these criteria. The Lestard studies provide comparatively broad cellular phenotyping and direct apoptosis measurements, whereas Elbe et al. broadened the number of cancer-cell lines but relied mainly on WST-1 viability. Ramírez-Rivera and Bernal, and Mehrafsar and Mokhtari provide molecular readouts, but with limited pathway validation. Park should be regarded as exploratory because of limited methodological validation, whereas the historical observations by Maman cannot be quality-assessed adequately because standardized experimental and acoustic data are unavailable. Thus, the present evidence base is better regarded as hypothesis-generating than confirmatory.

It was not until 2013 that the first peer-reviewed study came out investigating music exposure to cancer cells under standardized experimental conditions [2]. Lestard, Valente, Lopes and Capella exposed MCF-7 human breast cancer cells to musical compositions and analyzed for cell viability, cell cycle distribution, morphology and hormone-related cellular parameters [2]. They compared distinct recordings, but the design did not isolate any specific acoustic property. MCF-7 breast cancer cells were exposed to the first movement of Mozart’s “Sonata for two Pianos (D major, KV. 448)”, to the first movement of Beethoven’s “5th Symphony”, and to Ligeti’s “Atmosphères”. Authors found that Beethoven and Ligeti decreased the cell cycle up to cell death while Mozart did not. Instead, morphologic alterations, such as cell volume and granularity, changed in Beethoven´s and Ligeti´s music exposure but not in Mozart’s music. Particularly Beethoven’s music induced a significant reduction in cancer-cell volume. The recordings differed in potentially measurable properties such as sound-pressure level, frequency spectrum, temporal envelope and rhythm, but these variables were not independently controlled or quantified at the cell layer. The observed differences therefore cannot be directly attributed to a composer, genre, perceived harmony or any single acoustic parameter. This early study reported differences among music-exposed non-auditory cancer cells, while leaving the effective acoustic variables and causal mechanism unresolved. Lestard and Capella then provided a follow-up study in 2016 using the same music pieces in which they extended their analyses on another cancer-cell line, MDA-MB-231 with an apoptotic assay, using annexin V/propidium iodide flow cytometry and a cell migration assay [3]. After 30 min of music exposure and three days of whole study period, they found that Ligeti’s “Atmosphères”. induced the highest percentage of apoptotic cells and identified an increased phospho-p53 and cleaved-caspase-3 ELISA signal in MCF-7 cells. Conventional MCF-7 cells lack functional caspase-3 because of a CASP3 deletion [36]; the reported cleaved-caspase-3 signal therefore requires independent verification of cell-line identity and assay specificity. Thus, these marker changes do not establish a causal p53/caspase pathway without functional intervention. Instead, Beethoven’s “5th Symphony” and Mozart’s “Sonata” significantly diminished the migration of MDA-MB-231 cells. Authors could proof this only in breast cancer-cell lines but not in human erythroleukemia cell lines. The different responses across cell lines are suggestive of context dependence, but neither mechanical mediation nor cancer selectivity was causally tested. Overall, although authors employed a good method panel and study design, the experiments remain heterogenous and artificial.

Ramírez-Rivera and Bernal examined AGS gastric cancer cells exposed to metal and classical music, assessing proliferation and expression of apoptosis- and cell-cycle-related genes [37]. Proliferation increased in the metal music condition but not in the classical-music condition. Both exposures were associated with increased expression of caspase-3, caspase-8 and cyclin B1; p53 expression decreased with classical music, whereas PUMA expression decreased with metal music. These mixed findings do not support a uniform anticancer response. Changes in gene expression do not demonstrate caspase activation, apoptosis or a causal pathway without complementary functional assays. Moreover, the comparison does not isolate the responsible acoustic parameters, so the observations cannot be attributed to genre or perceived musical qualities. The study therefore extends the reported associations to gastric cancer cells while leaving reproducibility, mechanism and cancer selectivity unresolved [37].

Another kind of audible sound that was exposed to cancer cells was proposed by Mehrafsar and Mokhtari. These authors examined the effect of Quran recitation, in one experimental arm alone and in another arm along with cisplatin. They analyzed the cell viability, motility, and BCL2L12 (BCL2-like 12) gene expression on PC-3 human prostate adenocarcinoma cells [38]. Cell cultures were exposed for 2 h at a reported sound-pressure level of 90 dB. This was an in vitro exposure, not a patient listening protocol. Prolonged exposure to high sound levels may pose a hearing risk, depending on duration, spectrum and measurement weighting. The reported culture exposure therefore establishes neither a safe human listening dose nor therapeutic efficacy; clinical translation would require ear-level exposure assessment and auditory safety evaluation. Because BCL2L12 is involved in apoptosis-related regulation and has been associated with several malignancies, authors suggested that audible recitation may influence prostate cancer-cell proliferation and migration through apoptosis-related gene expression. Authors found a considerable inhibitory effect of Quran recitation on the proliferation and migration of PC-3 cells. Gene expression of BCL2L12 was significantly downregulated in PC-3 cells exposed to both cisplatin and Quran recitation. However, the observed BCL2L12 decrease is an association. Without targeted inhibition or rescue experiments, it is hypothetical that BCL2L12 mediates the reported cellular effects. The causal acoustic features remain unknown because the frequency spectrum, temporal modulation and mechanical exposure at the cell layer were not adequately characterized. From a study-quality perspective, the reported 90 dB exposure and 2 h duration provide more quantitative information than genre labels alone, but the acoustic spectrum and the mechanical dose actually transmitted to the culture surface were not sufficiently characterized. Moreover, the stronger effect observed with cisplatin complicates attribution of apoptosis-related changes specifically to the acoustic stimulus. The study therefore supports an association between recitation exposure and altered PC-3 behaviour, but not a defined sound-specific apoptotic mechanism.

Elbe and colleagues later investigated Turkish (19th and early 20th century) and western violin and piano classical music (mainly from the classic and romantic era) in several cancer-cell lines, including MCF-7 breast cancer, PC-3 prostate cancer, U87 glioblastoma, COLO741 colon cancer and SKOV-3 ovarian cancer cells [39]. Using WST-1 viability assays, they reported that repeated exposure to Turkish classical music, western classical piano music and western classical violin music reduced viability in several cell lines, particularly after two 40 min exposure periods separated by a 1 h interval at reported sound levels of 70–100 dB. Authors found reductions in MCF-7, PC-3, U87 and COLO741 cells, whereas SKOV-3 ovarian cancer cells showed increased viability after single exposures and less consistent suppression after repeated exposures. The significance of this study is that authors analyzed a cancer-cell-line-specific response. This is remarkable as it argues against a universal anti-cancer effect of music but rather suggests that cellular phenotype, tissue origin and mechanosensitivity of different cancer cells react differently. Importantly, the SKOV-3 findings should be treated as a biologically meaningful negative/pro-proliferative result rather than as an exception to be discounted. Increased viability after single exposures, together with the variable effects after repeated exposure, demonstrates that the direction of the response can reverse with cell type and exposure schedule. The broad 70–100 dB sound-level range also limits comparison among conditions because it does not specify spectral composition or vibration at the cell layer. Its upper end raises hearing-safety concerns if comparable levels reach a patient’s ears. These culture experiments do not establish a clinically acceptable exposure: the acoustic dose reaching tumour tissue in vivo and the dose at the ears would require separate assessment. Thus, this study strengthens evidence for heterogeneity but weakens any claim of a general music-induced anticancer response.

Recently, Park and colleagues examined SH-SY5Y neuroblastoma cells exposed to musical pieces differing in frequency and decibel characteristics at varying frequencies and decibel levels for 24 and 48 h. They exposed cells to the music pieces “River Flows in You” by Yiruma (41 dB), “Brandenburg Concerto No.3” by Bach (62 dB), and “This is What it Feels Like” by Armin Van Buuren (86 dB) [40]. In the 2D model, viability differed between the three musical exposures; however, frequency, sound level and other musical properties varied together. In the 3D model, the cell masses separated into smaller clusters, but this structural change does not establish cell death. The study also contains an important pro-survival finding: the lower-frequency/lower-level condition increased the number of viable cells in the 2D model. However, because the selected pieces simultaneously differed in reported decibel level, spectral content, rhythm and production characteristics, the experiment cannot separate a frequency effect from an intensity or composition effect. The 3D disaggregation endpoint should likewise not be equated automatically with cancer-cell death, because fragmentation or detachment of a tumour mass does not by itself demonstrate loss of clonogenic viability. These limitations substantially reduce the strength of causal inference.

A remarkable study comes from Kaivola and colleagues that does not directly deal with the effect of audible sound on cancer cells but with the effect of vocal fold movement through stretching and vibration on vocal fold cancer. The authors reported that mimicking physiological vocal fold movement through stretching or vibration reduced oncogenic β-catenin and YAP nuclear levels in patient-derived vocal fold cancer models [41]. Although this study was not designed primarily to assess music-induced cancer-cell death, it is relevant because it demonstrates that cancer cells from mechanically active tissues are mechanically sensitive and can respond to vibration-like stimulation through mechanotransduction pathways linked to malignancy. The study supports the broader concept that sound-related mechanical stimulation can alter cancer-cell behaviour, especially through YAP-/TAZ-related mechanobiology. Table 1 gives an overview on the relevant studies available, investigating the effect of audible sound and music on cancer-cell viability and cancer-cell death (Table 1).

The available evidence is limited and methodologically heterogeneous; no formal study-quality grade can be assigned. The confidence in studies is lower when conclusions rely predominantly on metabolic viability assays, limited gene panels, morphological observations, or poorly characterized acoustic exposures. None of the available studies provides a complete acoustic dose–response analysis, independent replication across laboratories, or in vivo validation. Equally important, the literature contains several neutral, inconsistent, or potentially pro-tumour findings: Mozart produced weaker or no comparable cytotoxic effects in the initial Lestard study; heavy metal was associated with increased proliferation in AGS cells; SKOV-3 cells showed increased viability under some exposure conditions; and lower-frequency music increased viable SH-SY5Y cells in the Park study. These observations argue against selective emphasis on anticancer outcomes and against a universal direction of the effect.

Taken together, the experimental studies report associations between music or audible-sound exposure and cancer-cell viability, phenotype, migration, gene expression and apoptosis-related markers in vitro. The Lestard studies provide comparatively detailed observations in breast cancer cells [2,3], while Elbe et al. examined several cancer-cell lines using mainly viability assays [38]. However, reproducible, causal and cancer-selective effects have not been established. Ramírez-Rivera and Bernal extend the evidence towards gene-expression responses in gastric cancer cells, while Mehrafsar and Mokhtari reported changes in prostate cancer-cell viability, migration, and BCL2L12 expression [37]. However, the marked heterogeneity of cell models, exposure systems, acoustic characterization, endpoints, and experimental quality precludes firm conclusions regarding a reproducible anticancer effect, and no available evidence demonstrates efficacy in vivo. Across studies, no consistent relationship can currently be established between cancer-cell outcome and a particular musical style or single physical sound parameter. Reported exposures span substantially different sound-pressure levels, durations, temporal structures, and spectral compositions, while most studies do not quantify the acoustic field or mechanical vibration reaching the actual cell layer. Consequently, apparent differences between Mozart, Beethoven, Ligeti, classical piano or violin music, heavy metal, electronic music, and recitation cannot presently be interpreted as genre-specific biological effects. Likewise, frequency, loudness, rhythm, harmonic structure, spectral complexity, and temporal modulation are interdependent features of musical stimuli and were generally not varied independently in the available studies. A rigorous comparison will therefore require standardized acoustic paradigms in which frequency spectrum, amplitude, temporal modulation, exposure duration, and mechanical coupling to the culture system are independently controlled.

Accordingly, audible sound and music should currently be regarded as experimental mechanobiological modulators rather than established anticancer interventions.

## 7. Limitation of Current Research

The evidence is limited by heterogeneous exposure systems, incomplete acoustic characterization and inconsistent biological endpoints. Frequency spectra, temporal modulation, sound pressure at the cell layer and mechanical coupling to culture vessels are rarely quantified, preventing reliable dose comparisons or attribution of findings to specific acoustic parameters. Reliance on metabolic viability assays or marker changes further limits inference about cell death and causal pathways.

Translation to intact tumours is uncertain because cell monolayers do not reproduce tissue architecture, stromal interactions or the absorption, scattering and attenuation of sound in vivo. Findings from ultrasound and other mechanical models cannot be directly extrapolated to audible sound. Reported outcomes include reduced, increased and unchanged viability. Together with the lack of independent replication and in vivo validation, this variability precludes claims of a reproducible, causal or cancer-selective anticancer effect.

## 8. Future Perspectives

Research priorities are calibrated exposure systems, sham controls and independent variation in frequency spectrum, sound-pressure level, temporal envelope and exposure duration. Measurements at the cell layer should document mechanical coupling and delivered exposure. Malignant and non-malignant cells, spheroids and patient-derived organoids should be compared using dose–response designs and complementary measures of apoptosis, long-term survival and migration. Mechanistic studies should combine calcium imaging, mitochondrial and ROS measurements, cytoskeletal analysis and apoptosis profiling with targeted inhibition, genetic perturbation or rescue experiments. These approaches should test candidate pathways, including PIEZO1, integrins, FAK and YAP/TAZ, specifically under audible-sound exposure. Transcriptomic and proteomic analyses may guide hypotheses, but functional validation is needed to distinguish causal mediators from accompanying marker changes.

## 9. Conclusions

Audible-sound and music studies report associations with cancer-cell outcomes in vitro, providing hypotheses for acoustic mechanobiology. However, the present evidence does not establish an anticancer intervention. Its scientific value lies in defining testable questions about how measurable acoustic exposures relate to cellular behaviour.

## Figures and Tables

**Table 1:** Overview of experimental studies investigating the effects of music, audible sound or vibration-like mechanical stimulation on cancer-cell viability, migration, apoptosis-related signalling and mechanobiological responses. The table summarizes the applied cell models, acoustic exposure conditions, methodological approaches and main findings reported in the available literature.

Author (Reference)	Cancer Model	Acoustic Exposure	Methods and Endpoints	Main Findings	Comment
Maman [4]	Uterine cancer cells; early microscopic observation.	Guitar, gong, xylophone and human voice.	Microscopic visual monitoring of cell morphology.	Unverified anecdotal description; no quantitative outcome.	Not peer-reviewed quantitative evidence; no documented controls or acoustic measurements.
Lestard [2]	MCF-7 breast cancer cells; in vitro.	Mozart piano “KV 448”, Beethoven “5th Symphony”, Ligeti “Atmosphères”.	Viability, cell cycle, morphology, hormone-related parameters; FACS and membrane protein assays.	Beethoven and Ligeti reduced cell-cycle progression and induced morphological changes; Mozart showed weaker/no comparable effects.	First standardized evidence in non-auditory cancer cells; decisive acoustic parameter remains unclear.
Lestard [3]	MDA-MB-231 breast cancer cells; comparison with erythroleukemia cells.	Ligeti, Beethoven and Mozart; 30 min exposure, 3-day assessment.	Annexin V/PI and migration assays; p53 and reported cleaved-caspase-3 ELISA signal in MCF-7 cells.	Ligeti induced most apoptosis; Beethoven and Mozart reduced migration in MDA-MB-231 cells.	Reported MCF-7 caspase-3 signal requires verification; marker changes do not establish causality.
Ramírez-Rivera [37]	AGS gastric cancer cells; in vitro.	Classic and romantic era piano music vs. “Cannibal Corpse” heavy metal; 12 h exposure.	Gene-expression analysis: cyclin B1, caspase-3/-8, p53; proliferation-related endpoints.	Metal exposure was associated with increased proliferation; both exposures increased caspase-3/-8 and cyclin B1 expression. Classical music reduced p53 expression; metal music reduced PUMA expression.	Interesting gene-expression study but limited mechanistic depth and no independent replication.
Mehrafsar [38]	PC-3 prostate adenocarcinoma cells; in vitro.	Quran recitation ± cisplatin; 2 h exposure, approx. 90 dB.	Viability, migration and BCL2L12 gene expression.	Recitation reduced proliferation and migration; combination with cisplatin had stronger inhibitory effects; BCL2L12 decreased.	BCL2L12 change is associative; functional mediation and relevant acoustic features remain untested.
Elbe [39]	MCF-7, PC-3, U87, COLO741 and SKOV-3 cells.	Turkish classical and western classical piano/violin music; 70–100 dB; single or repeated exposures.	WST-1 viability assays across multiple cell lines.	Repeated exposure reduced viability in MCF-7, PC-3, U87 and COLO741; SKOV-3 showed inconsistent or increased viability.	Shows cancer-cell-line-specific responses; argues against a universal anti-cancer music effect.
Park [40]	SH-SY5Y neuroblastoma cells; 2D and 3D models.	Yiruma 41 dB, Bach 62 dB, Armin van Buuren 86 dB; 24/48 h exposure.	2D viability assessment; 3D tumour mass detachment/disaggregation analysis.	2D viability differed among musical exposures; 3D masses separated into smaller clusters.	Frequency and level were confounded; 3D disaggregation did not demonstrate cell death.
Kaivola [41]	Patient-derived vocal fold cancer models.	Physiological stretching or vibration-like stimulation.	Analysis of mechanotransduction markers, especially nuclear beta-catenin and YAP.	Mechanical stimulation reduced oncogenic beta-catenin and YAP nuclear levels.	Not a music study but supports vibration-sensitive tumour mechanobiology.

FACS, fluorescence-activated cell sorting; ELISA, Enzyme-linked Immunosorbent Assay; WST-1, water-soluble tetrazolium assay; YAP, yes-associated protein. Study-quality interpretation: direct cross-study comparison is limited because acoustic dose, frequency spectra, exposure geometry, controls and endpoints were not standardized. Reported increases in proliferation/viability and null or inconsistent findings are considered equally relevant to reductions in viability and apoptosis when assessing the direction and reproducibility of the evidence.

## Data Availability

No new data were created or analyzed in this study. Data sharing is not applicable to this article.

## References

[1] Bradt J., Dileo C., Magill L., Teague A. (2016). Music interventions for improving psychological and physical outcomes in cancer patients. Cochrane Database Syst. Rev..

[2] Lestard Ndos R., Valente R.C., Lopes A.G., Capella M.A. (2013). Direct effects of music in non-auditory cells in culture. Noise Health.

[3] Lestard N.R., Capella M.A. (2016). Exposure to Music Alters Cell Viability and Cell Motility of Human Nonauditory Cells in Culture. Evid. Based Complement Altern. Med..

[4] Maman F. (1997). The Role of Music in the Twenty-First Century.

[5] Ingber D.E. (2003). Mechanobiology and diseases of mechanotransduction. Ann. Med..

[6] Jaalouk D.E., Lammerding J. (2009). Mechanotransduction gone awry. Nat. Rev. Mol. Cell Biol..

[7] Luo T., Mohan K., Iglesias P.A., Robinson D.N. (2013). Molecular mechanisms of cellular mechanosensing. Nat. Mater..

[8] Wood A.K., Sehgal C.M. (2015). A review of low-intensity ultrasound for cancer therapy. Ultrasound Med. Biol..

[9] Liaquat H., Al-Jumaily A.M. (2025). Resonance-Induced Therapeutic Technique for Skin Cancer Cells. Ultrasound Med. Biol..

[10] Del Rosario-Gilabert D., Valenzuela-Miralles A., Esquiva G. (2024). Advances in mechanotransduction and sonobiology: Effects of audible acoustic waves and low-vibration stimulations on mammalian cells. Biophys. Rev..

[11] Baumgartner K., Schleicher M.T., de Campos A.M., Taufer P., Engelke H., Westerhausen C. (2025). Actin Filament and Cell Orientation Align with Surface Acoustic Wave Propagation and Cell Migration in Vibration-Enhanced Wound Healing. ACS Appl. Mater. Interfaces.

[12] Sokabe M., Naruse K., Sai S., Yamada T., Kawakami K., Inoue M., Murase K., Miyazu M. (1997). Mechanotransduction and intracellular signaling mechanisms of stretch-induced remodeling in endothelial cells. Heart Vessel..

[13] Kang H., Hong Z., Zhong M., Klomp J., Bayless K.J., Mehta D., Karginov A.V., Hu G., Malik A.B. (2019). Piezo1 mediates angiogenesis through activation of MT1-MMP signaling. Am. J. Physiol. Cell Physiol..

[14] Lai A., Chen Y.C., Cox C.D., Jaworowski A., Peter K., Baratchi S. (2021). Analyzing the shear-induced sensitization of mechanosensitive ion channel Piezo-1 in human aortic endothelial cells. J. Cell Physiol..

[15] Cox C.D., Bae C., Ziegler L., Hartley S., Nikolova-Krstevski V., Rohde P.R., Ng C.A., Sachs F., Gottlieb P.A., Martinac B. (2016). Removal of the mechanoprotective influence of the cytoskeleton reveals PIEZO1 is gated by bilayer tension. Nat. Commun..

[16] Belousova P., Kwon S., Jung M.H., Kim K.S. (2026). Impact of mechanical property alterations on cancer cell motility and metastasis. Crit. Rev. Oncol. Hematol..

[17] Massey A., Stewart J., Smith C., Parvini C., McCormick M., Do K., Cartagena-Rivera A.X. (2024). Mechanical properties of human tumour tissues and their implications for cancer development. Nat. Rev. Phys..

[18] Schibber E.F., Mittelstein D.R., Gharib M., Shapiro M.G., Lee P.P., Ortiz M. (2020). A dynamical model of oncotripsy by mechanical cell fatigue: Selective cancer cell ablation by low-intensity pulsed ultrasound. Proc. Math. Phys. Eng. Sci..

[19] Heydarian A., Milani D., Beni H.M., Babaee M., Goudarzi H.R. (2026). How do vibration stimulation frequencies affect the nonlinear dynamics and mechanical characterization of breast cancer cells?. Biochem. Biophys. Rep..

[20] Ambattu L.A., Ramesan S., Dekiwadia C., Hanssen E., Li H., Yeo L.Y. (2020). High frequency acoustic cell stimulation promotes exosome generation regulated by a calcium-dependent mechanism. Commun. Biol..

[21] Federico G., Carotenuto A.R., Cutolo A., Palumbo S., Moccia M., Paladino S., Santoro M., Russo T., Fraldi M., Carlomagno F. (2025). Ultrasound-induced mechanical damage of cancer cell cytoskeleton causes disruption of nuclear envelope and activation of cGAS-STING. Sci. Rep..

[22] Anggayasti W.L., Imashiro C., Kuribara T., Totani K., Takemura K. (2020). Low-frequency mechanical vibration induces apoptosis of A431 epidermoid carcinoma cells. Eng. Life Sci..

[23] Heyden S., Ortiz M. (2016). Oncotripsy: Targeting cancer cells selectively via resonant harmonic excitation. J. Mech. Phys. Solids.

[24] Tijore A., Margadant F., Dwivedi N., Morgan L., Yao M., Hariharan A., Chew C.A.Z., Powell S., Bonney G.K., Sheetz M. (2025). Ultrasound-mediated mechanical forces activate selective tumor cell apoptosis. Bioeng. Transl. Med..

[25] Luo A.C., Qian Z., King M.R. (2026). Focused ultrasound for cellular mechanoactivation and nanomedicine delivery in cancer. BioMed Microdevices.

[26] Yu J., Hu J.R., Tian Y., Lei Y.M., Hu H.M., Lei B.S., Zhang G., Sun Y., Ye H.R. (2025). Nanosensitizer-assisted sonodynamic therapy for breast cancer. J. Nanobiotechnol..

[27] Datta P., Moolayadukkam S., Chowdhury D., Rayes A., Lee N.S., Sahu R.P., Zhou Q., Puri I.K. (2024). Recent Advances and Future Directions in Sonodynamic Therapy for Cancer Treatment. BME Front..

[28] Wang N., Luo L., Xu X., Zhou H., Li F. (2024). Focused ultrasound-induced cell apoptosis for the treatment of tumours. PeerJ.

[29] Sankar G., Roy Choudhury A., Luha R., Kumar A., Kulkarni K., Yao M., Pratap R., Kapali A., Tijore A. (2026). Selective killing of cancer-associated fibroblasts by ultrasound-mediated mechanical forces. Biomaterials.

[30] Rix A., Heinrichs H., Porte C., Leenaars C., Bleich A., Kiessling F. (2024). Ultrasound-induced immune responses in tumors: A systematic review and meta-analysis. J. Control. Release.

[31] Chen T., Chen J., Chen M., Song R., Wang M., Yu X. (2025). Acoustic immune reprogramming: A novel paradigm for spatiotemporally controlled immune regulation using ultrasound-responsive nanoplatforms. Front. Immunol..

[32] Du Y., Yang J., Xu A., Chen S., Fu D. (2025). Research progress on the induction of immunogenic cell death in tumor immunotherapy using a sonodynamic therapy nanoparticle delivery system. Front. Immunol..

[33] Kumeta M., Takahashi D., Takeyasu K., Yoshimura S.H. (2018). Cell type-specific suppression of mechanosensitive genes by audible sound stimulation. PLoS ONE.

[34] Kumeta M., Otani M., Toyoda M., Yoshimura S.H. (2025). Acoustic modulation of mechanosensitive genes and adipocyte differentiation. Commun. Biol..

[35] Lei Z., Jiang H., Liu J., Liu Y., Wu D., Sun C., Du Q., Wang L., Wu G., Wang S. (2023). Audible Acoustic Wave Promotes EV Formation and Secretion from Adherent Cancer Cells via Mechanical Stimulation. ACS Appl. Mater. Interfaces.

[36] Janicke R.U., Sprengart M.L., Wati M.R., Porter A.G. (1998). Caspase-3 is required for DNA fragmentation and morphological changes associated with apoptosis. J. Biol. Chem..

[37] Ramirez-Rivera S., Bernal G. (2019). Music Is Capable of Inducing Changes in Gene Expression in Gastric Cancer Cells. J. Gastrointest. Cancer.

[38] Mehrafsar A., Mokhtari M.J. (2018). Effect of Exposure to Quran Recitation on Cell Viability, Cell Migration, and BCL2L12 Gene Expression of Human Prostate Adenocarcinoma Cell Line in Culture. Health Spirit. Med. Ethics.

[39] Ceren Elbe M.O.O., Yigitturk G., Turan S.G., Elbe H. (2023). Cellular Response of Cancer to Music: Mirror, Mirror, on the Wall, Which is the Most Effective of Them All?. Med. Rec..

[40] Park J. (2024). Exploring the Impact of Music Frequency and Decibels on the Viability of Neuroblastoma Cancer Cells. Int. J. High Sch. Res..

[41] Kaivola J., Punovuori K., Chastney M.R., Abdo H., Follain G., Mathieu M., Joshi O., Miroshnikova Y.A., Krautgasser F., Di Franco J. (2026). Restoring the tumour mechanophenotype of vocal fold cancer reverts its malignant properties. Nat. Mater..

